# Health equity under siege: the populist challenge to women’s health in Europe

**DOI:** 10.1093/eurpub/ckaf266

**Published:** 2026-03-24

**Authors:** Rachel Greenley, Anneli Uusküla, Pia Kirkegaard, Marc Bardou, Martin McKee, CBIG-SCREEN Consortium, Marc Bardou, Berit Andersen, Pia Kirkegaard, Rikke Buus Bøje, Mette Tranberg, Rosa Legood, Anna Foss, Martin McKee, Rachel Greenley, Liu Sun, Paolo Giorgi Rossi, Luca Ghirotto, Letizia Bartolini, Giusy Iorio, Laura Bonvicini, Olivra Djuric, Noemi Auzzi, Paola Mantellini, Nuno Lunet, João Firmino-Machado, Ana Fernandes, Margarida Teixeira, Firmino Machado, Mariana Amorim, Inês Baía, Romeu Mendes, Cláudia Gouvinhas, Anneli Uusküla, Anna Tisler, Adriana Baban, Diana Tăut, Nicoleta Jiboc, Florian Nicula, Alexandra Tolnai, Iulia Gavrila, Rebecca Moore, Vanessa Moore, Heidi Ulrike Siller, Partha Basu, Isabel Mosquera Metcalfe, Keitly Mensah, Eric Lucas, Lise Rochaix, Camilla Fiorina, Violette Delisle, Irina Todorova, Yulia Panayotova, Tatyana Kotzeva, David Ritchie, Helena Ros Comesana, Meritxel Mallafré-Larrosa, Ginevra Papi, Christiane Dascher-Nadel

**Affiliations:** Department of Health Services, Research and Policy, London School of Hygiene and Tropical Medicine, London, United Kingdom; Institute for Family Medicine and Public Health, University of Tartu, Ravila, Tartu, Estonia; University Research Clinic for Cancer Screening, Randers Regional Hospital, Randers, Denmark; Inserm‐Centre d’Investigations Cliniques 1432 (CIC 1432), CHU Dijon‐Bourgogne, Dijon, France; Department of Health Services, Research and Policy, London School of Hygiene and Tropical Medicine, London, United Kingdom

Women in Europe have unmet needs for many preventive health services, exemplified by cervical cancer, where death rates vary at least sevenfold [1]. Despite being largely preventable through vaccination for human papillomavirus (HPV) and screening, structural barriers leave many women, such as migrants, ethnic minorities, and those in precarious circumstances, without access to services [2]. What progress has been achieved in vaccination uptake among girls and boys [3], introduction of self-sampling, and HPV testing replacing labour-intensive cytology [4], is now threatened by populist rhetoric spreading from the United States [5].The second Trump administration’s assault on science primarily targets women’s health programmes domestically [6], but the 2025 US National Security Strategy[7] and statements by Donald Trump[8] and his Vice President[9] have emboldened similar narratives in Europe. They frame preventive programmes as ‘ideological’ or ‘foreign’, rewrite legislation, and cut funding streams that dare to mention terms like diversity, women, gender, inclusion or vaccine confidence. This stifles innovation, weakens institutional capacity, and perpetuates inequities.

Safeguarding women’s health requires resilience. This requires acceptance that screening is a core element of public health, funding being diversified beyond vulnerable national budgets, and coalitions being built to defend health equity as a democratic principle. Without proactive measures, populist obstruction risks reversing hard-won gains and excluding Europe’s most vulnerable women from essential preventive care. As researchers who have documented the structural barriers facing many European women, we can identify three specific risks and outline ways to mitigate them.

1. Populist obstruction: U.S. rhetoric influences European policy through transnational networks, media amplification, and the adoption of similar frames by political parties. Populist actors block, delay, or dilute legislation, rarely opposing cancer prevention outright but framing resistance as protecting traditions, rejecting ‘foreign’ agendas, or curbing spending. The result is the same: vulnerable women remain excluded from lifesaving programmes. Legislative delays impede funding, hinder the adoption of new technologies, and weaken public health campaigns. Obstruction also creates uncertainty for providers and NGOs, discouraging long-term investment. In this way, populist resistance undermines institutional capacity and perpetuates health inequities among disadvantaged groups.
2. Funding vulnerability: Experience in the United States offers a cautionary example of how quickly gains can be reversed. Similar dynamics are already visible in parts of Europe, where screening reforms and vaccination initiatives have stalled following shifts in political leadership or budgetary priorities, contributing to persistent gaps in policy adoption and coverage across countries [10]. When populist or ideologically driven governments reallocate budgets, women’s health initiatives are often the first to face cuts, framed as “non-essential” or politically contentious. In the U.S., programs such as Title X family planning services and Planned Parenthood funding have repeatedly been targeted, with reductions justified under populist rhetoric about fiscal responsibility or moral values. These cuts disproportionately affect low-income and marginalised women, limiting access to preventive care including cancer screenings. The ripple effects are profound: clinics close, outreach programs shrink, and trust in public health systems erodes. For research projects in Europe, this American experience underscores the importance of diversifying funding sources, building coalitions that defend women’s health as a universal right, and anticipating political shifts that could destabilise program sustainability.
3. Delegitimisation: Populist rhetoric often portrays programmes as unnecessary, wasteful, or even dangerous, especially vaccination, undermining their legitimacy. For communities with negative past experiences and hesitancy toward healthcare, this stigmatisation reinforces mistrust and reduces participation. The impact is twofold: fewer women access care, and public health campaigns lose credibility. By politicising screening, populist narratives erode confidence in medical institutions and deepen health inequities among those most in need.

These risks demand urgent action, which we address through five imperatives.

1. We must anticipate how ideological opponents will frame attacks and respond effectively. Emphasising public health benefits for all, rather than a ‘women’s rights’ agenda, makes programmes harder to dismiss as partisan. Highlighting cancer prevention, cost-effectiveness, and population-wide benefits strengthens legitimacy. U.S. campaigns framed around broad health outcomes have proven more resilient than those tied to reproductive rights. In Europe, screening should be presented as routine preventive medicine to reassure hesitant communities and to help initiatives endure even in volatile political climates.
2. We must build broad coalitions with local NGOs, medical associations, and trusted community leaders, creating an inclusive network that champions women’s health, and form a distributed base of support that is harder to marginalise or defund. Such coalitions amplify advocacy, lend credibility, and make preventive programs harder for populist politicians to dismiss as ‘ideological’. By uniting diverse voices, including health professionals, civil society, and grassroots actors, we can build resilience, ensure continuity, and strengthen public trust. At the EU level, coalitions such as Europe’s Beating Cancer Plan and EU4Health unite member states, NGOs, and health professionals in ways that transcend national politics.
3. Communication must be deliberately inclusive and depoliticised, prioritising messages that emphasise health equity and the universal benefits of cancer prevention. Populist narratives are amplified through digital platforms that exploit algorithmic echo chambers, spreading misinformation rapidly and eroding trust in institutions; countering this requires partnerships with fact-checkers, investment in digital literacy, and using trusted voices on social media, supported by effective controls on social media companies. Communication strategies should avoid terminology that could be mischaracterised as ‘identity politics’, as this framing risks politicising public health interventions and eroding public trust. Instead, narratives should present prevention as cost-effective, evidence-based measures that safeguard population health. Inclusive communication strengthens legitimacy and resilience against ideological attacks by focusing on shared values and collective well-being.
4. Funding models should anticipate political volatility. They must be diversified, moving beyond reliance on national budgets, which are vulnerable to political shifts and populist retrenchment. Projects should pursue EU-level grants, philanthropic contributions, and cross-border collaborations to create a stable and diversified funding base independent of national governments. Such strategies reduce exposure to domestic policy volatility and safeguard continuity of preventive health programmes. By embedding financial pluralism into project design, initiatives can maintain momentum even in environments where populist actors seek to curtail women’s health spending.
5. Sustained progress in preventive health programs requires proactive engagement with the policy environment. This involves continuous monitoring of legislative developments at national and regional levels to identify emerging risks and opportunities. When misinformation or obstruction arises, rapid-response advocacy briefs should be prepared to provide clear, evidence-based counterarguments. Such timely interventions help maintain momentum, protect funding streams, and reinforce the legitimacy of prevention initiatives against politicisation and ideological challenges.

Addressing unmet needs in women’s health in Europe requires more than technical fixes; it demands political resilience. Populist narratives, amplified by U.S. rhetoric, threaten progress by delegitimising gender-specific health initiatives and obstructing policy and funding. Embedding strategies such as coalition building, inclusive communication, diversified funding, and proactive policy engagement frames preventive programmes as universal public health imperatives. Anticipating and countering ideological challenges safeguards equity, protects vulnerable populations, and upholds access to preventive care as a fundamental right rather than a political bargaining chip.

Conflict of interest: The authors all undertake research to improve women’s health, which could be adversely affected by policies pursued by radical populist politicians.

## Data Availability

No primary data were used in writing this paper.
